# Systematic Omics Analysis Review (SOAR) Tool to Support Risk Assessment

**DOI:** 10.1371/journal.pone.0110379

**Published:** 2014-12-22

**Authors:** Emma R. McConnell, Shannon M. Bell, Ila Cote, Rong-Lin Wang, Edward J. Perkins, Natàlia Garcia-Reyero, Ping Gong, Lyle D. Burgoon

**Affiliations:** 1 Oak Ridge Institute for Science and Education, Research Triangle Park, NC, United States of America; 2 National Center for Environmental Assessment, Office of Research and Development, United States Environmental Protection Agency, Arlington, VA, United States of America; 3 National Exposure Research Laboratory, Office of Research and Development, United States Environmental Protection Agency, Cincinnati, OH, United States of America; 4 United States Army Corps of Engineers, Department of the Army, Department of Defense, Vicksburg, MS, United States of America; 5 Department of Chemistry & Biochemistry, Jackson State University, Jackson, MS, United States of America; 6 SpecPro, Inc, Vicksburg, MS, United States of America; 7 National Center for Environmental Assessment, Office of Research and Development, United States Environmental Protection Agency, Research Triangle Park, NC, United States of America; West Virginia University, United States of America

## Abstract

Environmental health risk assessors are challenged to understand and incorporate new data streams as the field of toxicology continues to adopt new molecular and systems biology technologies. Systematic screening reviews can help risk assessors and assessment teams determine which studies to consider for inclusion in a human health assessment. A tool for systematic reviews should be standardized and transparent in order to consistently determine which studies meet minimum quality criteria prior to performing in-depth analyses of the data. The Systematic Omics Analysis Review (SOAR) tool is focused on assisting risk assessment support teams in performing systematic reviews of transcriptomic studies. SOAR is a spreadsheet tool of 35 objective questions developed by domain experts, focused on transcriptomic microarray studies, and including four main topics: test system, test substance, experimental design, and microarray data. The tool will be used as a guide to identify studies that meet basic published quality criteria, such as those defined by the Minimum Information About a Microarray Experiment standard and the Toxicological Data Reliability Assessment Tool. Seven scientists were recruited to test the tool by using it to independently rate 15 published manuscripts that study chemical exposures with microarrays. Using their feedback, questions were weighted based on importance of the information and a suitability cutoff was set for each of the four topic sections. The final validation resulted in 100% agreement between the users on four separate manuscripts, showing that the SOAR tool may be used to facilitate the standardized and transparent screening of microarray literature for environmental human health risk assessment.

## Introduction

Government agencies and environmental consultants develop human health risk assessments to determine the potential exposure and toxicity risks of chemicals, a process which involves consideration of all of the available published scientific literature on that chemical. Experts evaluate and integrate the studies that are available, make judgments on the quality of the science, and choose appropriate studies to derive cancer or noncancer toxicity reference values. A National Academy of Science Committee reviewing the draft Integrated Risk Information System (IRIS) Toxicological Review of Formaldehyde recommended that the IRIS Program develop “clear concise statements of criteria” when choosing studies to exclude or include for toxicity reference value calculations [1].

Significant work has been done by authors such as Fostel et al [2] and Schneider et al [3] to determine the criteria that are crucial for understanding the quality and reproducibility of toxicological studies in general. However, these criteria are not designed for use with transcriptomic studies, and are not adequate to provide an assessment of the entire study. Microarrays, one of many transcriptomic tools, are vastly different than the whole-animal toxicity studies that risk assessors and assessment teams are accustomed to evaluating. In acknowledgement of the complicated and varied procedures and analysis required to perform a microarray experiment, the gene expression microarray community created the “Minimum Information About a Microarray Experiment” (MIAME) [4] standard, along with data reporting requirements that have been adopted by several journals. Though this is a community standard for transcriptomic microarrays, it does not specifically consider their application to toxicogenomic studies for the purpose of human health risk assessment.

One method of combining the need to consider next generation technology with systematic approaches and transparency is through the development of a tool for “systematic reviews” of microarray literature. Systematic review methods are becoming increasingly more common, especially in medical and public health fields which involve a plethora of stakeholders and have wide-ranging human health implications [5]. A tool for performing such reviews would allow risk assessors and assessment teams to transparently apply standard criteria for judging the studies that they find in literature searches and include in their assessments. However, there are currently no systematic review tools focused on the applicability of toxicogenomic studies for use in human health risk assessment.

The Systematic Omics Analysis Review (SOAR) tool originated from our interest in developing a distributable tool to facilitate the systematic screening of transcriptomics studies using existing community standards as criteria, so that such studies can become more widely applied to risk assessment. The Toxicological Reliability Assessment (ToxR) Tool [3], MIAME standard [4], and the Checklist for Exchange and Interpretation of Data from a Toxicology Study [2] were resources for question development. After a spreadsheet of questions was generated, multiple rounds of testing were performed by scientists to refine and determine the appropriate weight for questions, and ultimately validate user agreement across a test set of published studies.

Our current implementation of SOAR is focused on screening/identifying transcriptomic studies that can be used to support a risk assessment. These aspects could include, but are not limited to: hazard identification, mode of action analyses, weight of evidence evaluations, assumptions, and read-across. Future work will focus on implementing a series of questions to assess dose-response studies to ascertain if they are suitable for benchmark dose modeling analysis.

## Methods

### Source of questions

The initial questions used to develop the SOAR tool were derived from three main peer-reviewed sources: 1) MIAME, 2) ToxRTool, and 3) the Checklist for Exchange and Interpretation of Data from a Toxicology Study. The questions that pertained directly to microarray data came from MIAME [4], while general questions on information needed for repeating a toxicological study are drawn from the ToxRTool [3] and the Checklist [2]. A few questions were also written based on expert guidance because they were not included elsewhere. The ToxRTool in particular was also used as a general guide for how to design and structure this type of tool.

### Development of the tool

Questions from the source materials were organized in a Google Drive Spreadsheet (see https://docs.google.com/spreadsheet/ccc?key=0AmmkQbxxSwwKdDNqYjBxaGhYTHFPX3NhaTMyT1A2WXc). A “Preliminary Questions” section was developed to screen out manuscripts that do not have three or more biological replicates or do not pertain to a chemical exposure and are thus not relevant to chemical risk assessment. This section also asked questions that determined the type of study (*in vivo, in vitro*, etc) in order to tailor the questions asked in the subsequent sections (answers to these questions do not affect the score). The remaining questions were organized into five sections: 1) Test System (including separate sets of questions for in vivo and in vitro studies), 2) Test Substance, 3) Experimental Design, 4) Microarray Data, and 5) Suitability for Benchmark Dose (BMD) modeling, as seen in Table 1. Each question had a “yes” or “no” answer, with a few questions also containing a “Not Applicable” option. Initially weights were set to one for every question, with an “NA” answer causing the weight to drop to zero. After testing weights were adjusted to range from 0 to 1 depending on the importance of the information, as determined by participating microarray experts.

**Table 1:** Question sections included in the original version of the SOAR tool compared to the final version.

Question sections		Original # of Questions	Final # of Questions
	Preliminary Questions	4	5
I.	Test System (in vivo human, in vivo non-human, or in vitro)	7–10	3–10
II.	Test Substance	6	6
III.	Experimental Design	11	5
IV.	Microarray Data (either including raw data or not)	18	5–8
V.	Suitability for Benchmark Dose Modeling	12	-

The section "Test System" has different questions based on the type of study. The maximum number of questions a paper can require is 34, though only 29 of them would be scored. The first five basic questions are used to exclude inappropriate papers and to set up the questions required, and are therefore not given a score.

The spreadsheet format allowed for the use of drop-down response menus, automatic calculation of weighted scores for each section of questions, sections for rater comments, and automated scripts that adjust the questions that users were presented based on the type of data in the study, as well as automatic bibliographic data entry. Additionally, *mouse-over* comments were added to the spreadsheet to provide more information and examples of how to find the answer to the question within a published manuscript. Questions were edited first internally using a training set of four manuscripts, shown in Table 2, for which the pass/fail designation was determined *a priori* [6]–[9]. During the course of testing, some questions were re-worded for clarity, other questions were removed because the evaluation team did not find them informative, and the weights of the questions were adjusted to better reflect their importance in determining suitability for use in an assessment.

**Table 2:** The papers used to develop and test the SOAR tool. The first four were used only during internal development of the questions.

	ID	Reference	PMID	Study Type	Study Compound	Rounds used
Papers Used During Internal Development		Fertuck et al. [6]	12915738	In vivo, mouse	ethynylestradiol	Development
		Permenter et al. [9]	22110744	In vitro, rat	nickel, chromium, cadmium	Development
		Frericks et al. [8]	18691609	In vitro, mouse	TCDD	Development
		Fracchiolla et al. [7]	21296121	In vitro, human	TCDD	Development
Papers Used for General Question Editing and Formatting	1	Woods et al. (2009) [11]	19376150	In vitro, mouse	Hypochlorous acid	Round 1 (n = 3); Round 2 (n = 4)
	2	Chen et al. [12]	18230668	In vivo, zebrafish	Retinoic acid, TCDD	Round 1 (n = 3); Round 2 (n = 4)
	3	Kong et al. [13]	19951294	In vivo, Drosophila	Ethanol	Round 1 (n = 4); Round 2 (n = 2)
	4	Pedersen et al. [14]	17597826	In vivo, human	Nickel	Round 1 (n = 5); Round 2 (n = 2)
	5	Nilsson et al. [15]	22570695	In vivo, rat	Multiple pesticides, plastics, TCDD, and jet fuel	Round 1 (n = 4); Round 2 (n = 3)
	6	Song MO, et al. [16]	19549813	In vitro, human	Copper	Round 1 (n = 4); Round 2 (n = 3)
	7	Boyle et al. [17]	20179299	In vivo, human	Cigarette Smoke	Round 1 (n = 2); Round 2 (n = 5)
	8	Carolan et al. [18]	17108109	In vivo, human	Cigarette Smoke	Round 1 (n = 2); Round 2 (n = 5)
Papers Used for Targeted Question Editing	13	Andreasen et al. [19]	16443690	In vivo, zebrafish	TCDD	Round 3 (n = 3)
	14	Song R, et al. [20]	19095052	In vivo, human	PBDEs	Round 3 (n = 4)
	15	Gottipolu et al. [21]	19165385	In vivo, rat	Diesel exhaust	Round 3 (n = 3)
	16	Heiden et al. [22]	17884332	In vivo, zebrafish	TCDD	Round 3 (n = 5)
	17	Dreij et al. [23]	20382639	In vitro, human	Benzo[a]pyrene diol epoxide	Round 3 (n = 3)
	18	Suvorov et al. [24]	20056577	In vivo, rat	BDE-47	Round 3 (n = 5)
	19	McHale et al. [25]	19162166	Epidemiological	Benzene	Round 3 (n = 4)
Papers Used for Validation	9	Stevens et al. [26]	18192680	In vivo, mice	Diesel exhaust	Round 4 (n = 6)
	10	Gebel et al. [27]	20133372	In vivo, mice	Cigarette Smoke	Round 4 (n = 6)
	11	Landi et al. [28]	18297132	Epidemiological	Cigarette Smoke	Round 4 (n = 6)
	12	Hirano et al. [29]	21887816	In vitro, human	PAHs	Round 4 (n = 6)

Papers 1–8 were used by seven experts (internal and external) for 2 rounds of revising the questions. The last 11 were used by the same group to validate the tool and determine inter-rater reliability. Papers were chosen by performing a broad literature search and removing any that were affiliated with the expert in this study.

### Testing

Seven scientists with diverse backgrounds and experience with toxicogenomic data were recruited to assist in assessing and validating the SOAR tool (see Table 3 for details on participants) over the course of four separate rounds conducted over nine weeks. During the first two rounds, the scientists were asked to focus on editing, clarifying, reformatting, or suggesting questions for removal. In the third round statistics on user agreement were calculated to focus on improving the wording and the weights of the questions, specifically where users disagreed. In the fourth and final round, six of the experts rated the same four manuscripts (n = 6; one scientist dropped out of the study before this round) to validate the tool. Because of the small sample size throughout the study, percent agreement between users on the final pass/fail outcome for a manuscript was the only statistic used.

**Table 3:** Experts who participated in editing and validating the SOAR tool, their affiliations, and expertise.

Expert Name	Affiliation	Expertise
Shannon Bell	ORISE Fellow at NHEERL, USEPA, Research Triangle Park, NC	Systems biology, large data analysis
Lyle Burgoon	NCEA, USEPA, Research Triangle Park, NC	Systems biology, bioinformatics, data mining, risk assessment
Ila Cote	NCEA, USEPA, Arlington, VA	Risk assessment
Natalia Garcia-Reyero	Mississippi State University, Starkville, MS	Ecotoxicogenomics
Ping Gong	Badger Technical Services, Vicksburg, MS	Ecotoxicogenomics
Emma McConnell	ORISE Fellow at NCEA, USEPA, Research Triangle Park, NC	Ecotoxicology and environmental health, risk assessment/management
Edward Perkins	USACE, Vicksburg, MS	Toxicogenomics
Rong-Lin Wang	NERL, USEPA, Cincinnati, OH	Genomics, bioinformatics, data mining

Papers used for testing and evaluating SOAR were identified by performing a PubMed (http://www.ncbi.nlm.nih.gov/pubmed) literature search using the search string: microarray AND exposure. Nineteen papers were chosen that were pertinent to risk assessment of chemicals and not coauthored by the participating scientists. Papers were assigned to participants so that each paper was rated at least twice in one round and no participant rated the same paper more than once (n = 2–5 per paper per round, n = 6–7 per paper total; see Table 4 for exact sample sizes per paper per round).

**Table 4:** Results from Round 4 of testing.

		Scores by Author					
		EM	SB	RW	PG	LB	NGR
Paper 9	I. Test Organism (In vivo)	97	97	83	100	97	97
	II. Test Substance	100	100	82	100	100	100
	III. Experimental Design	100	100	100	100	100	100
	IV. Microarray Data	85	85	85	85	85	85
	Final Result:	**PASS**	**PASS**	**PASS**	**PASS**	**PASS**	**PASS**
Paper 10	I. Test Organism (In vivo)	80	97	97	90	93	97
	II. Test Substance	100	100	100	100	100	100
	III. Experimental Design	87	100	100	100	100	100
	IV. Microarray Data	96	92	96	92	92	85
	Final Result:	**PASS**	**PASS**	**PASS**	**PASS**	**PASS**	**PASS**
Paper 11	I. Human Subjects (In vivo)	69	81	69	100	100	81
	II. Test Substance	100	100	69	100	100	69
	III. Experimental Design	67	77	77	77	77	77
	IV. Microarray Data	81	75	38	69	62	38
	Final Result:	**FAIL**	**FAIL**	**FAIL**	**FAIL**	**FAIL**	**FAIL**
Paper 12	I. Test System (In vitro)	-	-	100	100	-	-
	II. Test Substance	-	-	94	94	-	-
	III. Experimental Design	-	-	87	87	-	-
	IV. Microarray Data	-	-	39	25	-	-
	Final Result:	**FAIL**	**FAIL**	**FAIL**	**FAIL**	**FAIL**	**FAIL**

Though some authors disagreed on specific answers to certain questions, the disagreement was not significant enough to change the final outcome for the papers. Paper 9 and 10 passed; paper 11 and 12 failed. For paper 12, EM, SB, LB, and NGR failed the paper in the “Basic Questions” section based on a lack of sufficiently biological replicates (tool requires n≥3), and therefore the following question sections were not answered. RW and PG did complete all the question sections, however, the paper still failed.

At the beginning of each round, the scientists were given PDF copies of their assigned manuscripts for that round with author, affiliation, date, and journal information removed. The participants were also given PDF copies of information pertaining to the raw data (e.g. a print out of the manuscript's entry in the Gene Expression Omnibus (GEO, http://www.ncbi.nlm.nih.gov/geo/) database), also with author and date information removed. Table 2 gives reference information for the papers used for testing. Participants were given approximately 10 days to answer all of the questions in the tool for the four papers in a round. After all participants had completed a round, feedback was collected on questions to edit, remove, or add and changes were made accordingly. The weighting was also modified and a pass/fail threshold was developed based on participant feedback.

## Results

### Round 1 & 2 (General Question and Format Editing)

Significant changes were made to the tool after the first two rounds of question adjustment. The number of questions dropped from a maximum of 61 questions to a maximum of 34 questions, as shown in Table 1. Several subjective questions were removed from the tool, along with questions that did not come from a peer-reviewed source. Originally there were 11 questions from MIAME, 23 questions from ToxRTool, 5 questions from Fostel et al [2], 12 questions from the Benchmark Dose Technical Guidance document [10], and 17 questions from domain experts. Section 5 pertaining to BMD modeling was removed because it required highly specific questions about the data and a level of understanding and time commitment beyond what should be expected from a first pass screening tool. That is not to say that SOAR cannot address the applicability of dose-response transcriptomic data. In fact, the questions in SOAR deal with those aspects of toxicogenomic (specifically transcriptomic) studies that are generally applicable. Rather, we are stating that at this time those types of questions are best left to BMD specialists (we do have future work that will specifically address BMD given our experiences here; thus, further discussion of BMD is outside the scope of this particular manuscript). Additionally, after the participating scientists rated a paper that involved human subjects (papers #4, 7, and 8), it became apparent that a separate set of questions was needed specifically for human studies under the “Test System” section. Originally the “Test System” questions were broken up into in vivo and in vitro but did not consider the human subject. With guidance from the participating scientists, a section was added for “In vivo, human” test subjects.

Finally, the “Microarray Data” section was split into two different sets of questions depending on whether or not raw data were available for the study. Less information is needed about how the normalized data were processed if interested scientists can access the data in raw form. After making these revisions, the final version of the tool involved five main sections with the first section setting up the tool and the remaining four sections used to score the paper. The final version contained 11 questions from MIAME, 19 questions from the ToxRTool, 4 questions from Fostel et al [2], and 6 questions from domain experts (if a question was repeated in two of the guidance sources it was only cited as being from one of the two).

Throughout the editing process the weights of the questions were also set. It was determined that a paper would be recommended for further consideration in a human health risk assessment if it received a score of at least 80% for each section.

### Round 3 (Targeted Question Editing)

**Figure 1:**
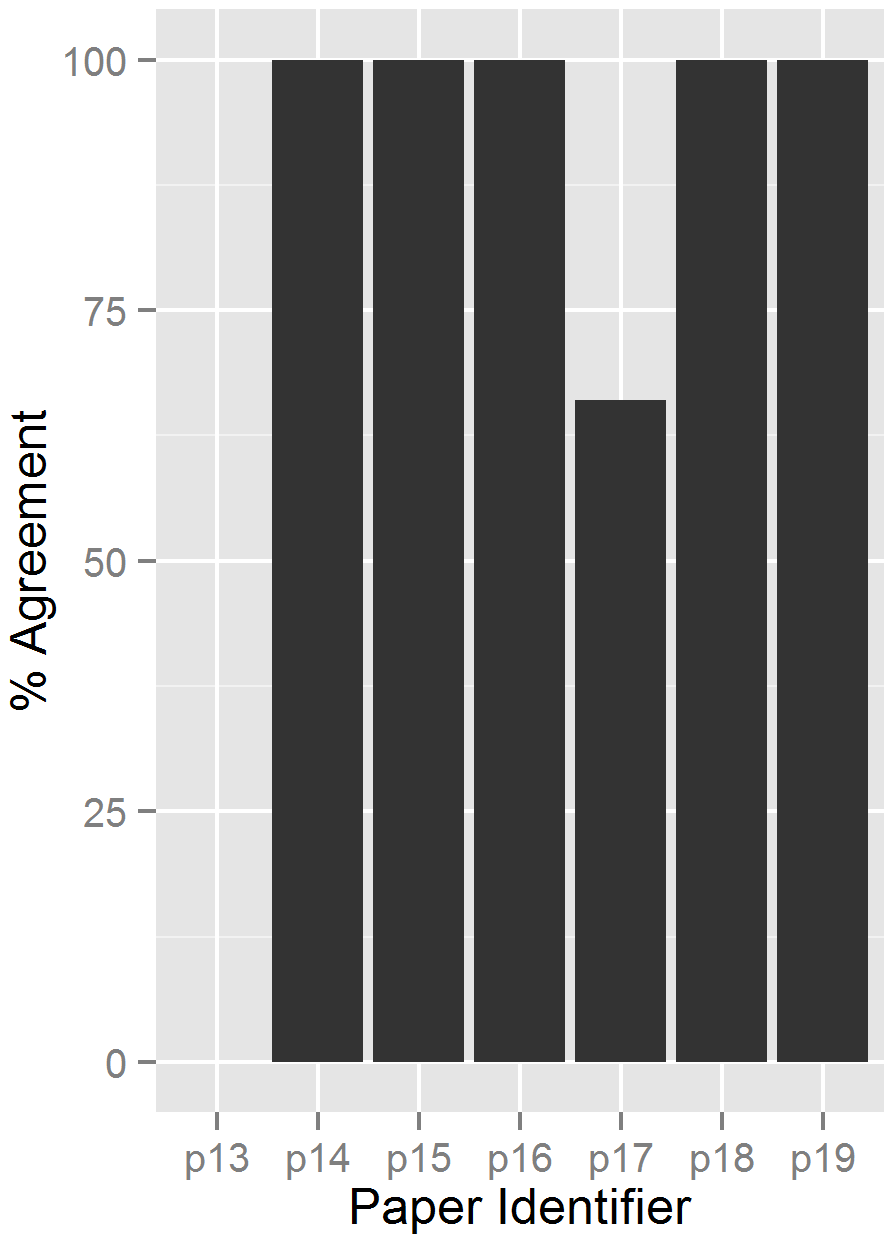
Percent agreement between experts on final pass/fail result of papers tested in Round 3. Each paper was tested through the SOAR tool by 3–5 expert experts. Paper 13 had no agreement between the three experts due to misunderstanding of the data presented in the paper. Paper 17 had 1 of 3 experts disagree.

Results from the third round of testing are shown in Fig. 1. Of the seven papers tested in this round, there were only two where the experts disagreed on the pass/fail outcome (i.e., there was not a unanimous pass/fail determination). For paper 13 there was no agreement between the three experts rating this paper, though further inquiry showed that this was caused by rater misunderstanding of the presented data. One scientist had incorrectly interpreted the study as being in vitro, while the other two answered as in vivo. Of the two scientists who determined it was an in vivo study, one failed it by answering “no” to the question “II.4. Is frequency and duration of exposure to the test substance explained?” while the other scientist answered “yes.”

For paper 17, two of the three experts were in agreement that the paper should fail. The third expert did not agree, making the percent agreement 66%. The main disagreement was on the answer to the question: “Are the study endpoint(s) and their method(s) of determination clearly described?” which may be considered subjective to some users.

### Round 4 (Validation)

Round 4, where all scientists rated the same papers as validation, produced 100% agreement on the final outcome (pass/fail) of all 4 papers (n = 6), as shown in Table 4. Concordance was achieved only after discussing the responses of one participant. The results were reviewed when there was disagreement on the pass/fail status of a paper. Each response given by the scientist who disagreed was examined and it was discovered that the scientist had incorrectly answered a single question that caused Papers 9 and 10 to fail (question II. 4: “Is frequency and duration of exposure to the test substance explained?”). The frequency and duration information was pointed out in the manuscript to the scientist who had answered “no.” This scientist realized that they missed this information while rating the manuscript and chose to revise their response, bringing their results into concordance with the rest of the group. Though there was some other disagreement between answers to specific questions for all of the papers, none of the differences were significant enough to change the pass/fail outcome of the tool.

The final questions included in the manual are provided in Table 5. For access to the full version of the tool, see the https://docs.google.com/spreadsheet/ccc?key=0AgWXniu3KhthdEhCcXdUMFVTeF9LVnZ1TFpJNkxZdEE=sharing.

**Table 5:** The full questions included in the SOAR manual and the source of the question if it was taken from an existing publication.

Preliminary Questions	Does the microarray experiment include biological replicates such that there is an n = 3?
	Is there reason to believe that data in this study could be useful in a chemical risk assessment?
	Is the microarray portion of the study performed in vivo or in vitro?
	Is the genetic material used in the microarrays taken from humans in vivo?
	Are raw data available for each hybridization?
Test Organism (In Vivo)	Is the species and/or strain of the test organism given? (ToxRTool)
	Is the sex of each animal given (if embyro, answer "NA")? (ToxRTool)
	Is the supplier of the animal given? (Fostel 2007)
	Is the days of acclimation given?
	Is age or body weight at the start of the study given of the test organisms? (ToxRTool)
	Is the number of animals per exposure group given?
	Is the route or method of administration of the test substance given? (ToxRTool)
	Is the euthanasia method given (including anesthetics, if used)?
	Is the tissue of origin given for each microarray sample? (MIAME)
	Are necessary information on housing and care conditions given such that the experiment could be repeated? (ToxRTool)
Human Subjects (In Vivo)	Is the sex of each human volunteer given? (ToxRTool)
	Is the number of volunteers per exposure group given?
	Is the route or method of exposure of the test substance given? (ToxRTool)
	Is the tissue of origin given for each microarray sample?
	Is all other necessary information on human volunteers given (see comment)?
Test System (In Vitro)	Is the species and/or strain of the source organism given for each cell line or primary cell culture used? (ToxRTool)
	Is the supplier of the sample given (answer "Yes" for primary cell culture)? (ToxRTool)
	Are necessary information on test system properties, and on conditions of cultivation and maintenance given such that the experiment could be repeated? (ToxRTool)
Test Substance	Is the test substance identified by name, chemical structure, or CAS number? (ToxRTool)
	Is the purity of the substance given? (ToxRTool)
	Is information given on the source/origin of the substance? (ToxRTool)
	Is frequency and duration of exposure to the test substance explained? (ToxRTool)
	For the test substance, are all dose concentrations and their units given? (ToxRTool)
	Is all information on the physico-chemical properties of the test item given that is necessary for judging the data? (ToxRTool)
Experimental Design	Are the study endpoint(s) and their method(s) of determination clearly described? (ToxRTool)
	Is the study design chosen appropriate for obtaining the substance-specific data aimed at? (ToxRTool)
	Is the method of RNA extraction for the microarray given?
	Are appropriate controls (vehicle, etc) included? (ToxRTool)
	If a two color array is used, did the author perform a dye swap?
Microarray Data (Raw Data Available)	Is it easy to discern the sample annotation for each raw data file (eg: which dose, which time point, which replicate)? (MIAME)
	Are there data available in the study that could be used to relate the exposure level from the microarray data back to the phenotype?
	Are microarray technical replicates used? (MIAME)
	Are the technical replicates clearly defined and easily identified? (MIAME)
	Did the author perform a confirmatory assay (such as qPCR)?
Microarray Data (No Raw Data Available)	Are final processed microarray data (normalized data) available for the study? (MIAME)
	Does the author clearly define all of the pre-processing methods that were applied to the microarray data? (MIAME)
	Do the authors include their methods for analyzing the data? (MIAME)
	Do the authors use published data analysis methods? (MIAME)
	Are there data available in the study that could be used to relate the exposure level from the microarray data back to the phenotype?
	Are microarray technical replicates used? (MIAME)
	Are the technical replicates clearly defined and easily identified? (MIAME)
	Did the author perform a confirmatory assay (such as qPCR)?

Not every question will be answered for every manuscript, given variation in the methods (in vivo, in vitro, etc). See https://docs.google.com/spreadsheet/ccc?key=0AgWXniu3KhthdEhCcXdUMFVTeF9LVnZ1TFpJNkxZdEE=sharing for a link to a publicly available version that includes weights applied to the questions, possible answers, and comments that provide more detail for each question.

## Discussion

The SOAR tool was designed to provide a transparent method for risk assessors and assessment teams to determine the suitability of specific, published microarray data for risk assessment purposes. The goals are similar to those of the ToxRTool but with a focus on issues of data analysis and study design specific to transcriptomic microarrays. The tool was developed through four rounds of testing with experts who have microarray experimental design and analysis experience. This repetitive testing allowed for a thorough evaluation of the wording, the appropriateness, and the weights applied to each question, as well as the general ease of use of the spreadsheet format. By the final validation round, all six experts agreed on whether the four papers would pass or fail.

The tool should be used by at least two different assessors familiar with microarray data for each manuscript being scored. If the two raters cannot agree on whether the manuscript passes or fails the tool, a third assessor should be consulted to make the final determination on the manuscript. The final round of validation was performed with this method in mind. Specific answers were examined only when an expert did not agree with the pass/fail designation of the rest of the group, as we would expect to occur in actual use. The situation discussed in the results of the validation, where one user made an honest mistake in their response that caused the papers to incorrectly fail, is a prime example of how multiple users will ensure the accuracy of the scores. Choosing to only have such comprehensive discussions when there was disagreement on the ultimate pass/fail result of the paper removed the need to discuss every question in the tool when the overall outcome was the same and benefited the users by reducing the overall length of time spent considering the literature.

Notably, there are disadvantages to taking such a broad look at the results. The main concern is that all users could make mistakes on a single paper that would result in an incorrect pass/fail designation. This could occur if the mistakes were made on the same question or on different questions. Additionally, these “mistakes” could occur in two different ways: 1) as the result of typing the incorrect response (choosing “no” for a question when the user meant to choose “yes”), or 2) as the result of differing interpretations of the questions or of the information in the manuscript being rated. If only the overall pass/fail result is examined in a case where multiple users make “mistakes,” both users may end up having incorrectly passed or failed a study. The remedy for this, which was also performed in the present study, was to have one person quickly compare the individual results from multiple users. Then, if answers differed on questions with high weights or on a significant number of questions, regardless of the final pass/fail designation, these can be brought to the attention of users.

Using repetitive testing with the same group of experts can result in the experts being trained in the meaning of the questions. By the final round their agreement in scoring may have been based on their collective understanding of the meaning of the questions and not on the innate clarity of the wording. This could mean that the tool would not produce such concordant results with new users who have less experience with the questions. In order to combat this issue, the majority of the questions were given comments in the spreadsheet with an alternate wording or clarifying details. Training would need to be provided for risk assessors to familiarize themselves with microarrays and their data, so specific training on the SOAR tool could be provided at that time.

The ultimate goal for the SOAR tool is to use natural language processing to enable computers to perform the first pass screen of all papers resulting from a literature search. If the computer gives a manuscript a “pass” then it will be sent to a human for further consideration and potential analysis. Many questions that could be considered subjective were removed by the final round of testing in an attempt to make the transition to natural language processing easier. Since this is a screening activity, we would need to ensure that the computer is more inclusive than exclusive, meaning we are more accommodating of including false positives and to build the models to ensure we have very few false negatives. There are questions on data quality that computers will not be capable of answering in the foreseeable future and these were set aside for human consideration after the first pass screening has taken place. As a result, the tool does not examine some of the more important aspects of data quality, such as overall reproducibility of the results. However, the goal is that after using the tool, risk assessors will be much better informed on the details of the paper and the study, as well as possible weaknesses and strengths so that they can make a final decision on whether or not it is appropriate to include in their assessment.

It is important to note that the results from the tool are not meant to be used as a strict cut-off; the opinion of an experienced expert should always take precedence over the result of the tool, which is intended only to make the process of identifying suitable studies more systematic and transparent. However, if agencies and risk assessors employ the SOAR tool, the information and the record created by collecting that information will be a critical step in fulfilling the need for transparent and thorough decisions on the quality of the omics studies.
